# Protective effects of ectoine on heat-stressed *Daphnia magna*

**DOI:** 10.1007/s00360-014-0860-x

**Published:** 2014-09-16

**Authors:** Bownik Adam, Stępniewska Zofia, Skowroński Tadeusz

**Affiliations:** 1 Department of Physiology and Ecotoxicology, Faculty of Biotechnology and Environmental Sciences, The John Paul II Catholic University of Lublin, Kontstantynów 1 „I”, 20-708 Lublin, Poland; 2 Department of Biochemistry and Environmental Chemistry, Faculty of Biotechnology and Environmental Sciences, The John Paul II Catholic University of Lublin, Kontstantynów 1 „I”, 20-708 Lublin, Poland

**Keywords:** Ectoine, *Daphnia magna*, Heat stress, Heat shock proteins

## Abstract

Ectoine (ECT) is an amino acid produced and accumulated by halophilic bacteria in stressful conditions in order to prevent the loss of water from the cell. There is a lack of knowledge on the effects of ECT in heat-stressed aquatic animals. The purpose of our study was to determine the influence of ECT on *Daphnia magna* subjected to heat stress with two temperature gradients: 1 and 0.1 °C/min in the range of 23–42 °C. Time to immobilisation, survival during recovery, swimming performance, heart rate, thoracic limb movement and the levels of heat shock protein 70 kDa 1A (HSP70 1A), catalase (CAT) and nitric oxide species (NOx) were determined in ECT-exposed and unexposed daphnids; we showed protective effects of ECT on *Daphnia magna* subjected to heat stress. Time to immobilisation of daphnids exposed to ECT was longer when compared to the unexposed animals. Also, survival rate during the recovery of daphnids previously treated with ECT was higher. ECT significantly attenuated a rapid increase of mean swimming velocity which was elevated in the unexposed daphnids. Moreover, we observed elevation of thoracic limb movement and modulation of heart rate in ECT-exposed animals. HSP70 1A and CAT levels were reduced in the presence of ECT. On the other hand, NOx level was slightly elevated in both ECT-treated and unexposed daphnids, however slightly higher NOx level was found in ECT-treated animals. We conclude that the exposure to ectoine has thermoprotective effects on *Daphnia magna*, however their mechanisms are not associated with the induction of HSP70 1A.

## Introduction

Temperature affects the biology of ectothermal organisms. Hyperthermia may cause alterations in growth, metabolism, reproduction, protein denaturation and formation of reactive oxygen species leading to oxidative stress (Pinkhaus et al., 2007). Some organisms react to thermal stress and other stressful factors by changing the composition of membrane lipids and by selective intracellular accumulation of low molecular weight substances, known as compatible solutes which protect proteins against denaturation without interference with cellular processes. As a result of accumulation of these solutes, the cells maintain osmotic balance and avoid water loss and subsequent irreversible dehydration (Roessler and Müller, 2001; Pastor et al., 2013). Compatible solutes can be divided into several structural groups: sugars (trehalose, sucrose), polyols (glycerol, sorbitol, mannitol, α-glucosyl-glycerol, mannosyl-glycerol, mannosyl glyceramide), *N*-acetylated diamino acids (like *N*-acetylglutaminylglutamine amide), betaines (such as glycine betaine and derivatives), amino acids (proline, glutamate, glutamine, alanine, ectoine and hydroxyectoine) and derivatives (Pastor et al., 2013).

Almost all compatible solutes increase thermal stability of proteins, however this protective effect under in vitro conditions is obtained after use of high concentrations. Trehalose is accumulated in yeast during heat stress for protection of enzymes against denaturation (Singer and Lindquist, 1998). Its protective ability at high temperatures (even boiling) was also observed in archaea and mammals (Santos and da Cota 2002). Ectoine (ECT) (1,4,5,6-tetrahydro-2-methyl-4-pyrimidine carboxylic acid) is produced by aerobic, chemoheterotrophic and halophilic bacteria to survive under extreme conditions (Galinski et al., 1985; Nagata and Wang 2001). Microorganisms such as *Marinococcus* sp. ECT1 synthesise and accumulate intracellular ectoine in response to osmotic stress in a hyperosmotic environment (Wei et al., 2011). Some studies showed that ECT protects bacterial cell membranes, enzymes and nucleic acids against hyperthermia by its accumulation within the cell wall. ECT was demonstrated to enhance stability of phytase, lactate dehydrogenase (LDH) and phosphofructokinase (PFK), enzymes sensitive to heating, urea, freezing and drying, freeze-thaw treating (Lippert and Galinski, 1992; Göller and Galinski 1999; Knapp et al., 1999; Zhang et al., 2006). It has been proposed that ECT increases the hydration of the cell surface, and thus increasing the mobility of the lipid head groups and fluidizing the lipid layer. The increased fluidity may be of advantage for cell membranes to cope with extreme conditions like temperature or osmotic pressure and may accelerate repair mechanisms in some cells (Harishchandra et al. 2010).

Although the protective effect in halophilic bacteria, human skin, cells and molecules has been described, there is a lack of knowledge about the effects of ectoine on heat-stressed animals in vivo. *Daphnia magna* are microcrustacean organisms subjected to temperature seasonal fluctuations with increasing temperature during summer. As poikilotherms they may increase their metabolism in response to higher temperature which also may make them more sensitive to various toxic substances such as heavy metals (Heugens et al., 2003). *Daphnia magna,* as a poikilothermic organism, is very sensitive to temperature alterations and its behavioural, physiological and biochemical changes induced by heat stress or possible thermoprotection may by determined by various methods. This microcrustacean is also a common laboratory animal model with transparent body. This unique feature gives a possibility to observe temperature-induced changes of physiological processes in a non-invasive way. Therefore, the purpose of our research was to determine the influence of ECT on the behavioural, physiological and biochemical level in *Daphnia magna.* The following endpoints were determined in ECT-exposed and unexposed daphnids subjected to high temperature at different gradients: time to immobilisation, survival during recovery, swimming performance, heart rate, thoracic limb movement and the levels of heat shock protein HSP70 1A, catalase and nitric oxide species.

## Materials and methods

### Culture method and ECT preparation

*Daphnia magna* were cultured for several generations in 6 L tanks with 5 L of aerated culture medium on the window ledge in a laboratory under light:dark period of 16:8 h. *Daphni*a culture medium was prepared following the ASTM standards (American Society of Testing and Materials, 1986). The medium was synthetic freshwater (48 mg of NaHCO_3_, 30 mg of CaSO_4_·2H_2_O, 30 mg of MgSO_4_ and 2 mg of KCl per litre of deionized water adjusted to a pH of 7.4), with a temperature of 23 ± 2 °C. The number of cultured daphnids was about 30 animals per litre. The animals were fed once daily with a few drops of powdered *Spirulina* (2 mg/L water) per tank and supplemented with a few drops of baker’s yeast (10 mg/L per tank). Feeding was stopped 24 h before the experiments.

Pure ECT standard (Sigma-Aldrich) of ≥95 % purity produced by *Halomonas elongata* was diltuted in *Daphnia* culture medium and used at concentrations of 2.5, 4, 20 and 25 mg/L. The concentrations were chosen on the basis of our previous observations on ECT toxicity in *Daphnia magna* and Material Safety Data Sheet for ectoine (Sigma-Aldrich). Our 50 mg/L turned out to be toxic to *Daphnia magna* in acute toxicity tests, therefore lower concentrations were used to avoid ECT toxicity. Neonates <24 h old of 2nd–5th generations were pre-treated with ECT for 24 h before each assay. Daphnids that were not treated with ECT were maintained in clean medium only.

### Immobilisation during heat stress and survival during recovery

10 daphnids were placed individually in 10 glass beakers containing 100 ml of one of the following aqueous solutions: 2.5, 4, 20 or 25 mg/L of ECT before the tests. One beaker with 1 individual in a medium only was also used. After 24 h of the exposure, 10 beakers of appropriate solution of ECT each containing 1 daphnid was subjected to heat stress in a programmable incubator (Salvislab, Incucenter IC80) with a built-in calibrated thermometer with the accuracy of 0.1 °C. Two separate heat stress experiments were performed in two temperature gradients: 0.1 °C/min (slower) and 1 °C/min (faster) used previously by Kivuori and Lahdes (1996) for determination of heat stress in *Daphnia magna*. Those two different gradients were used for comparison of possible thermoprotective effects of ECT in two different increasing temperature regimes. Separate heating experiment (repeated three times) was performed for each concentration of ECT and non-treated animals. During the experiments, the animals were observed for non-typical swimming behaviour, repeated measurements of the time and temperature values of daphnid immobilisation were recorded. Animals with ceased swimming movements for at least 15 s were treated as immobilised. After immobilisation of all daphnids in the heated experimental group subjected to the gradient of 1 °C or 0.1 °C/min, the crustaceans were immediately—all the immobilised and unimmobilised daphnids from the experimental groups—transferred to clean medium for recovery and were monitored for possible mortality by analysis of heart beat under a microscope. The number of survived and dead daphnids was counted after 15 min, 1, 3, 6, 12 and 24 h of recovery period.

### Swimming velocity

Swimming behaviour of heat-stressed *Daphnia magna* neonates was analysed according to the experimental setup described by Shimizu et al. (2002) with some modifications. 10 daphnids were transferred from the culture tanks to one of the observation culture dishes of 35 mm diameter containing 3 ml of the appropriate concentration of ECT. Each concentration of ECT was done in triplicate. Swimming velocity of the unexposed daphnids maintained in clean medium only and subjected to heat stress was also determined. The dishes were covered and placed in a programmable incubator (Salvislab, Incucenter IC80) with a built-in calibrated thermometer and subjected to heat stress with the temperature gradient of 1 °C or 0.1 °C/min. Moreover, control daphnids swimming in medium only at a temperature of 23 °C were treated as the control. Swimming behaviour of the ECT-exposed, unexposed and control animals in all observation dishes was video recorded for a minimum of 1 min (with resolution of 30 frames/s) with a digital camera Nikon D3100 mounted on a stand over the observation dish and processed with motion analysis software, Tracker^®^, version 4.82. The stand with the camera was placed inside the incubator in order to continue the temperature gradient during video recording. Vertical movement of *Daphnia* was negligible because of very small depth of the solution present in the observation dish. The video file with the recorded trajectories of swimming *Daphnia* was analysed frame-by-frame with Tracker^®^. By clicking with the cursor on *Daphnia* image in separate frames, the program plotted the whole trail left by a single *Daphnia* (interpreted by the program as a mass point) measuring its maximal, minimal and mean velocity (v) expressed in millimeters per second (mm/s). Since the animals moved virtually only in two dimensions swimming behaviour analysis was based on the trajectory represented by x and y coordinates. The velocities of ten daphnids calculated by software were plotted in the separate graphs which were then superimposed. Since swimming speed was not equal for all individuals in each experimental group and the control, the mean velocity (v) of 10 daphnids from each experimental group was treated as one result.

### Heart rate and thoracic limb activity

10 neonate daphnids were placed separately in ten 1.5-ml Eppendorf tubes and were subjected to 0.1 or 1 °C/min temperature gradient. Daphnids were taken in triplicate for optical measurement of physiological parameters: heart rate and thoracic limb movement at appropriate temperature. A single daphnid was transferred in a 50-µl droplet of appropriate concentration of ECT or clean medium in case of the control group to a microscope slide placed in a miniature microscope heated plate to maintain the appropriate temperature of the droplet. Temperature of the droplet was monitored by the use of an infrared thermometer with the accuracy of 0.2 °C. The daphnid was immediately put into measuring position and its movements were limited by cotton wool fibres placed on the microscope slide. The microscopic view of the examined daphnid was recorded for at least 2 min (with the speed of 30 frames per second) with a digital camera Nikon D3100 mounted on a light microscope. The magnification (30–100x) and camera resolution allowed performing the analysis with a good visibility of the heart and thoracic limbs. Heart rate and thoracic limb movement was analysed with Tracker^®^ software by a frame-by-frame method.

### Heat shock protein, HSP70 1A

Ten daphnids exposed to ECT at concentrations of 25 and 4 mg/L and ten unexposed individuals subjected to heat stress with the temperature gradient of 1 °C or 0.1 °C/min were taken for determination of HSP70 1A level when the temperature reached 30 °C or 40 °C. Daphnids from each experimental group were homogenised, in a pestle homogenizer, with 300 µl of Phosphate Buffered Saline (PBS). The resulting suspension was sonicated with ultrasonic homogenizer (Omni Ruptor 4,000) to disrupt the remaining cells and cell membranes. Afterwards, homogenates were centrifuged at 5,000×*g* for 5 min. The supernatants were taken for the analysis of HSP70 1A (HSP70 1A is referred to heat shock protein of molecular weight-70 kDa) level with a spectrophotometric assay (USCN, USA). This test is an ELISA sandwich immunoassay in which enzymatic colour reaction is linked to antibody specific to HSP70 1A. The test was performed according to the manufacturer’s manual. Briefly, 100 µl of experimental samples was added to the appropriate 96-well antibody-coated microplate in triplicate. The plate was covered with the plate sealer and incubated at 37 °C for 2 h. The liquid from each well was removed and 100 µl of detection reagent A (Biotin-conjugated polyclonal antibody) working solution was added to each well. The plate was incubated at 37 °C for 1 h after covering with the plate sealer. The solution was aspirated and washed with wash solution. The plate was then aspirated, inverted and blotted against the absorbent paper. 100 µl of detection reagent B (horseradish peroxidase) was added to each well and the plate was incubated for 30 min at 37 °C. Aspiration and wash was repeated 5 times and 90 µl of TMB (3,3′,5,5′-tetramethylbenzidine) for horseradish peroxidase detection was added to each well and the microplate was incubated for 20 min at 37 °C with protection from light. Next, 50 µl of stop solution (sulphuric acid) was added to each well and the solution was mixed. The microplate was then read with a spectrophotometric microplate reader (Biorad 550) at 415 nm.

### Catalase activity

Catalase activity was determined with a spectrophotometric method described by Goth (1991) with its slight modification to a micromethod. Briefly, ten heat-stressed (with the temperature gradients of 1 °C or 0.1 °C/min) individuals from experimental and control groups were taken at 30 and 40 °C and homogenised in a pestle micro-homogenizer with 300 µl of phosphate buffer. The homogenised samples were centrifuged, and 100 µl of supernatants were transferred to a 96-well microplate and incubated with 100 µl of 60 µM H_2_O_2_/60 mM sodium−potassium buffer at room temperature for 60 s in a 96-well microtiter plate at room temperature. The enzymatic reaction was stopped by the addition of 100 μL of ammonium molybdate (32 mM), and the absorbance of the yellow molybdate/hydrogen peroxide complex was measured with a spectrophotometric microplate reader (Biorad 550) at 415 nm. All samples were done in triplicate. Mixture of ammonium molybdate and the buffer was treated as blank.

### Nitric oxide species (NO_x_)

Nitric oxide (NO) has a short half-life, therefore analysis of NO_2_ which is a stable end product of nitric oxide metabolism, was used as a surrogate marker of NO. Briefly, ten heat-stressed (with the temperature gradients of 1 °C and 0.1 °C/min) and non-stressed daphnids from the experimental and the control groups were taken at 30 and 40 °C and washed in artificial medium, then dried on a paper towel and homogenised in a pestle micro-homogenizer in 300 µl of PBS. The suspension was sonicated with ultrasonic homogenizer (Omni Ruptor 4,000) to disrupt the remaining cells and cell membranes. Afterwards, homogenates were centrifuged at 5,000×*g* for 5 min. After centrifugation, 100 µl of supernatant was taken and placed in a well of a 96-well microplate. The wells from each experimental and control groups were done in triplicate. NO_x_ level was measured by the Griess reaction (Griess 1879; Smith et al., 2000) by adding 50 µl of 1 % sulfanilamide in 5 % H_3_PO_4_ to each well and subsequently 50 µl of naphthylethylenediamine dihydrochloride in distilled water. The microplate was incubated at room temperature for 15 min and the absorbance was read with a spectrophotometric microplate reader (Biorad 550) at 550 nm.

Results are presented as mean ± standard deviation (SD). All data were assessed for homogeneity of variance for ANOVA assumptions. Experimental data were analysed using ANOVA followed by Tukey’s test to detect differences among means. All analyses were completed using Delve^®^ statistical software. Values were statistically significant when *p* < 0.05.

## Results

### Time to immobilisation during heat stress

**Fig. 1:**
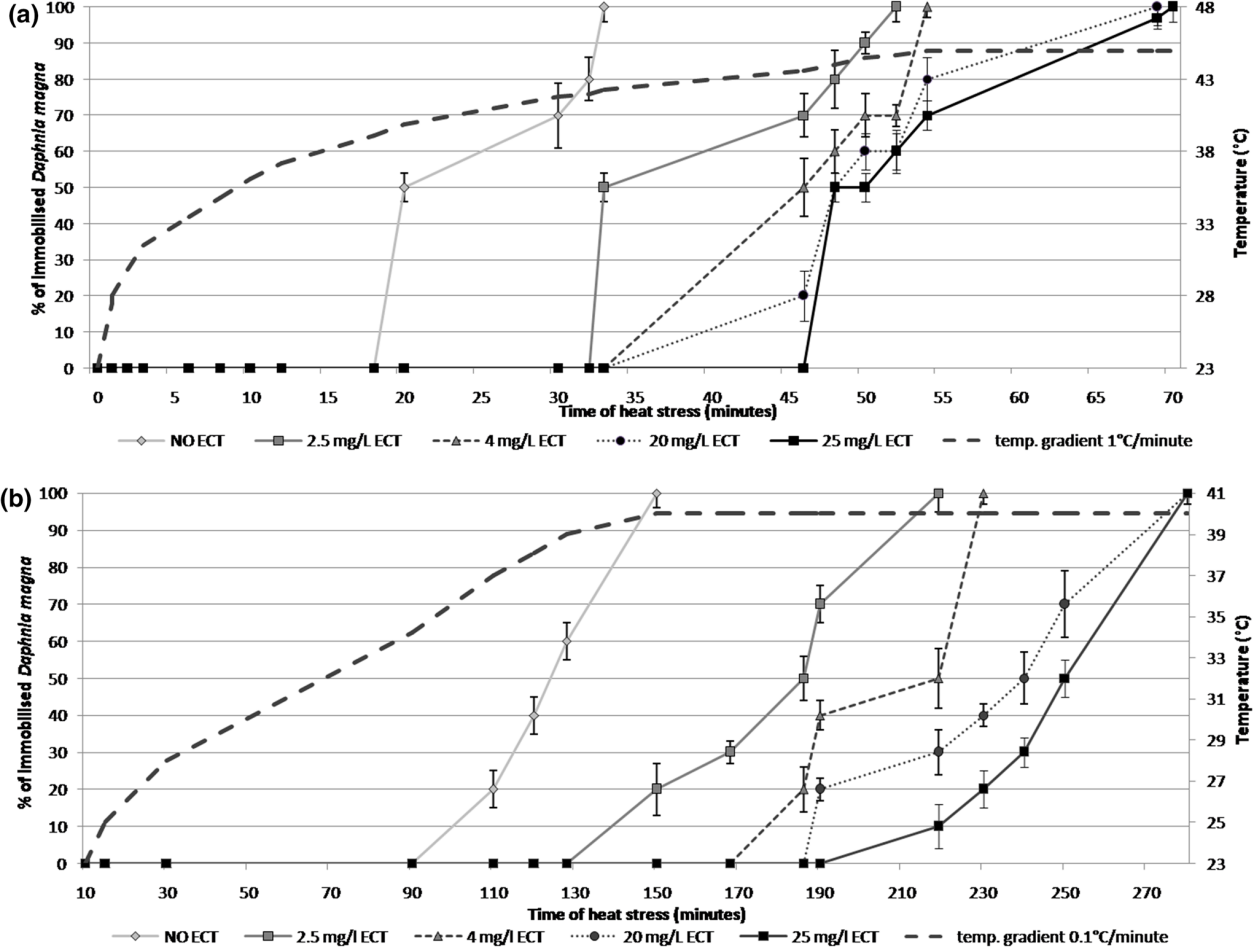
Time of immobilisation of *Daphnia magna* neonates immersed in different concentrations of ECT subjected to heat stress with two temperature gradients: 1 °C (**a**) and 0.1 °C/min (**b**). *Dotted lines* show temperature gradients. The results are presented as mean ± SD from 3 replicates, *n* = 30

The results showed that ECT prolonged time to immobilisation of daphnids subjected to heat stress in both temperature gradients (Fig. 1a). The present study showed that daphnids subjected to heat stress in both temperature gradients showed longer times until immobilisation in the presence of ECT in comparison to the unexposed crustaceans. 50 and 100 % immobilised animals, which were not treated with ECT was observed after 20 and 33 min of stress with the temperature gradient pf 1 °C/min, respectively. Heating with the temperature gradient of 1 °C/min resulted in immobilisation of 50 and 100 % of animals which were not treated with ECT after 20 and 33 min at temperature of 41.2 and 41.9 °C, respectively. (Figure 1a). Daphnids exposed to ECT at 2.5, 4, 20 and 25 mg/L showed 50 % immobilisation after 33, 46, 48 and 50 min at a temperature of 42.5 °C, 43.6 °C, 44.2 °C and 44.4 °C of heat stress, respectively and 100 % immobilisation was noted after 52, 54, 69 and 70 min at a temperature of 44.7, 44.9 and 45 °C, respectively. The animals treated with ECT and subjected to heat stress with the slower temperature gradient (0.1 °C/min) also showed much longer time to immobilisation compared to the unexposed ones (Fig. 1b). 50 % of immobilisation in the group of unexposed daphnids was observed after 125 min and 100 % daphnids were immobilised after 150 min of heating. Daphnids exposed to 2.5, 4, 20 and 25 mg/L of ECT showed 50 % immobilisation after 186, 219, 240 and 250 min, at respectively, when temperature reached 40 °C. 100 % immobilisation of microcrustaceans treated with 2.5, 4, 20 and 25 mg/L of ECT was noted after 219, 230, 250 and 280 min at a temperature of 40 °C, respectively.

### Survival during recovery after heat stress

**Fig. 2:**
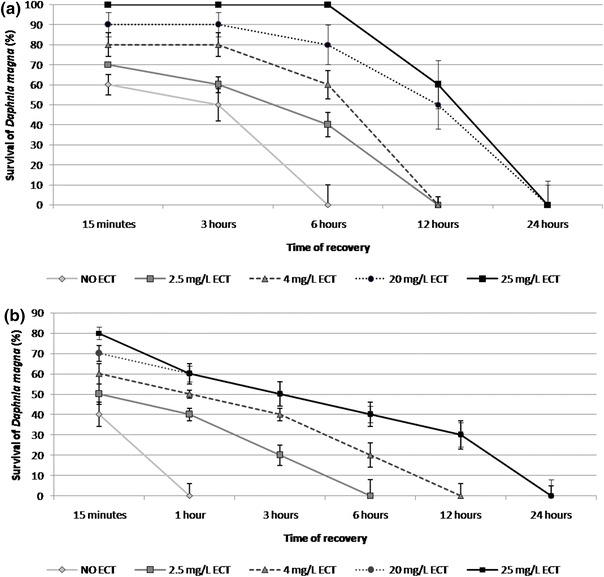
Survival of *Daphnia magna* during recovery of immobilised neonates exposed to various concentrations of ectoine after heat stress with the gradient of 1 °C/min (**a**) and 0.1 °C/min (**b**). The results are presented as mean ± SD from 3 replicates, *n* = 30

Mortality of daphnids previously subjected to heat stress was observed at different times of recovery period. 100 % of the individuals that were not exposed to ECT but subjected to heat stress with the gradient of 1 °C/min were dead after 6 h of recovery (Fig. 2a). Longer survival time was observed in daphnids previously exposed to ECT during heat stress. 100 % mortality of daphnids exposed to 2.5 and 4 mg/L was noted after 12 h of recovery. The longest survival time (100 % mortality was found after 24 h of recovery) was found in the animals exposed to 25 mg/L. Recovery experiment showed that heat stress with the gradient of 0.1 °C/min resulted in longer survival times of ECT-exposed daphnids in comparison to the unexposed animals (Fig. 2b). 100 % mortality of the unexposed daphnids was noted at 1 h of recovery time. No survived daphnids previously treated with ECT at 2.5, 4, 20 and 25 mg/L were found at 6, 12 and 24 h of recovery period, respectively.

### Swimming performance

**Fig. 3:**
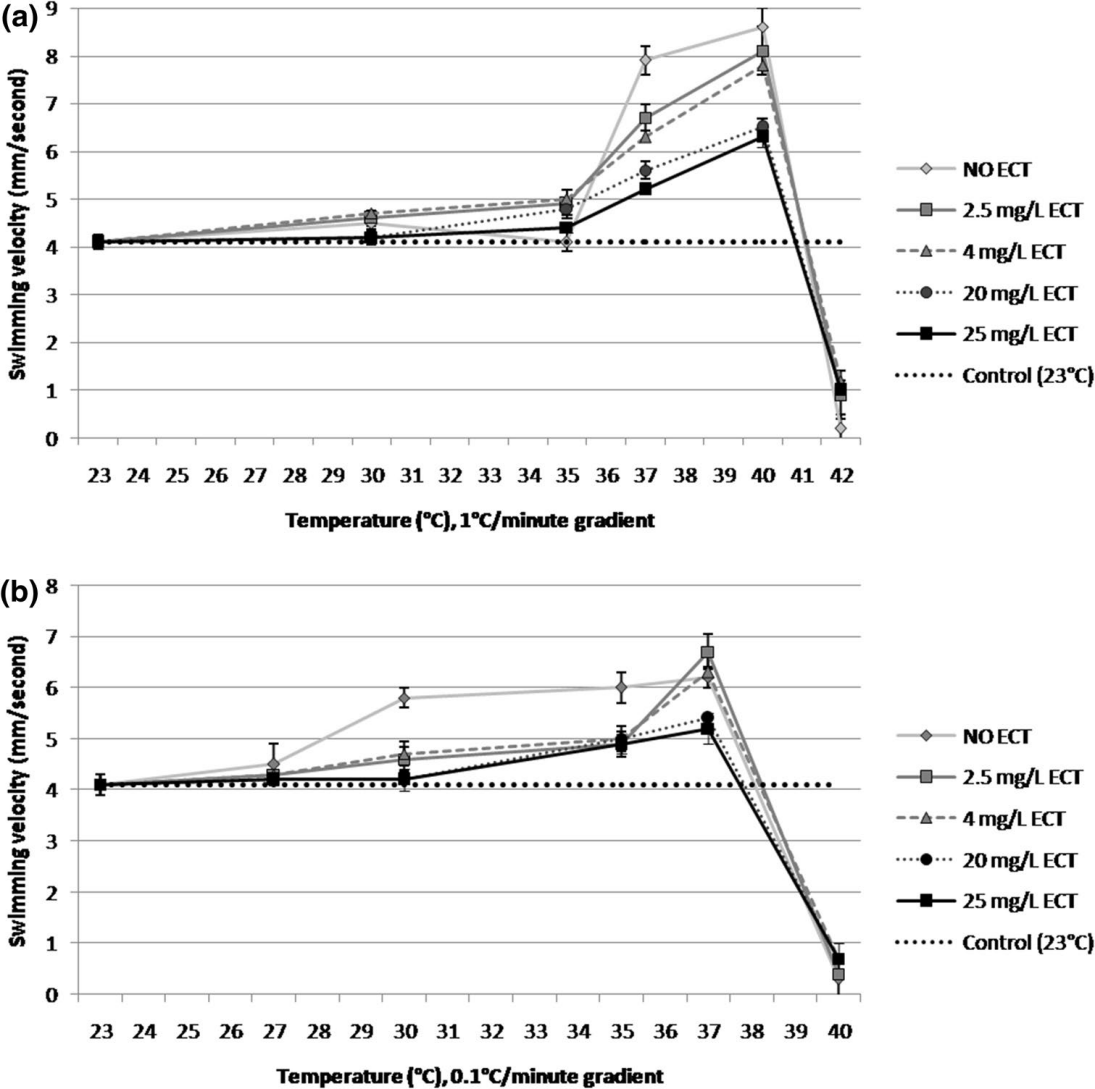
Swimming velocity of *Daphnia magna* exposed to various concentrations of ECT and subjected to heat stress with two gradients: 1 °C (**a**) and 0.1 °C/min (**b**). The *dashed*, *horizontal line* represents mean velocity for control daphnids (at 23 °C) which were not heat stressed and not ECT-exposed. The results are presented as mean ± SD, *n* = 30

The effects of ECT on swimming performance of heat-stressed daphnids are presented in Fig. 3. *Daphnia magna* that were not immersed in ECT but subjected to heat stress with the temperature gradient of 1 °C/min showed significantly increased mean swimming velocity at 37 °C (8 ± 0.3 mm/s) and 40 °C (8.5 ± 0.4 mm/s) when compared to the control group (4.2 ± 0.4 mm/s) (Fig. 3a). Behavioural changes in the heated daphnids were also noted. When the temperature increased to 37 °C, the individuals moved rapidly in all directions. Daphnids presented circle trajectories with increasing temperature to 40 °C. The mean velocity started to drop rapidly prior to immobilisation at a temperature above 40 °C with the magnitude of 0 ± 0.3 mm/s at 42 °C. On the other hand, ECT-exposed daphnids showed less significant changes of swimming behaviour during heat stress, in a concentration-dependent manner. The lowest increase of mean velocity at a temperature of 37 °C (5.2 ± 0.2 mm/s) and 40 °C (6.2 ± 0.3 mm/s) was observed in daphnids exposed to ECT at a concentration of 25 mg/L. Although mean swimming velocity of those animals began to decrease with the increasing temperature, 40 % of daphnids retained their swimming activity with mean velocity of 1.2 ± 0.3 mm/s. Less significant attenuation of the increase of mean velocity was observed in heat-stressed individuals with the temperature gradient of 1 °C/min and exposed to 4 and 2.5 mg/L of ECT. Figure 3b presents the effect of ECT on swimming velocity of heat-stressed daphnids with the temperature gradient of 0.1 °C/min. Heated animals that were not exposed to ECT showed increasing velocity at a temperature range from 30 °C (5.8 ± 0.2 mm/s) to 37 °C (6.2 ± 0.2 mm/s). Further increase of the temperature resulted in a rapid decrease of daphnid mean velocity to 0 ± 0.5 mm/s. ECT-exposed daphnids did not react to raising temperature by stimulation of their swimming speed compared to the unexposed animals. The individuals exposed to 20 and 25 mg/L of ECT showed most significant attenuation of the motility increase. Mean velocity of those daphnids was elevated at 35 °C (4.9 ± 0.2) and at 37 °C (5.2 ± 0.3 mm/s) and its rapid decrease was observed at 40 °C (0.5 ± 0.1 mm/s). Less significant attenuation of swimming speed changes induced by raising temperature were noted in daphnids exposed to lower concentrations of ECT and mean velocities at 37 °C were higher than those of the unexposed daphnids with magnitudes of 6.2 ± 0.3 and 6.5 ± 0.2 mm/s at 4 and 2.5 mg/L of ECT, respectively. Mean velocity of daphnids exposed to the two lower concentrations of ECT at 40 °C dropped rapidly and were 0.5 ± 0.06 and 0.5 ± 0.1 mm/s, respectively.

### Heart rate

**Fig. 4:**
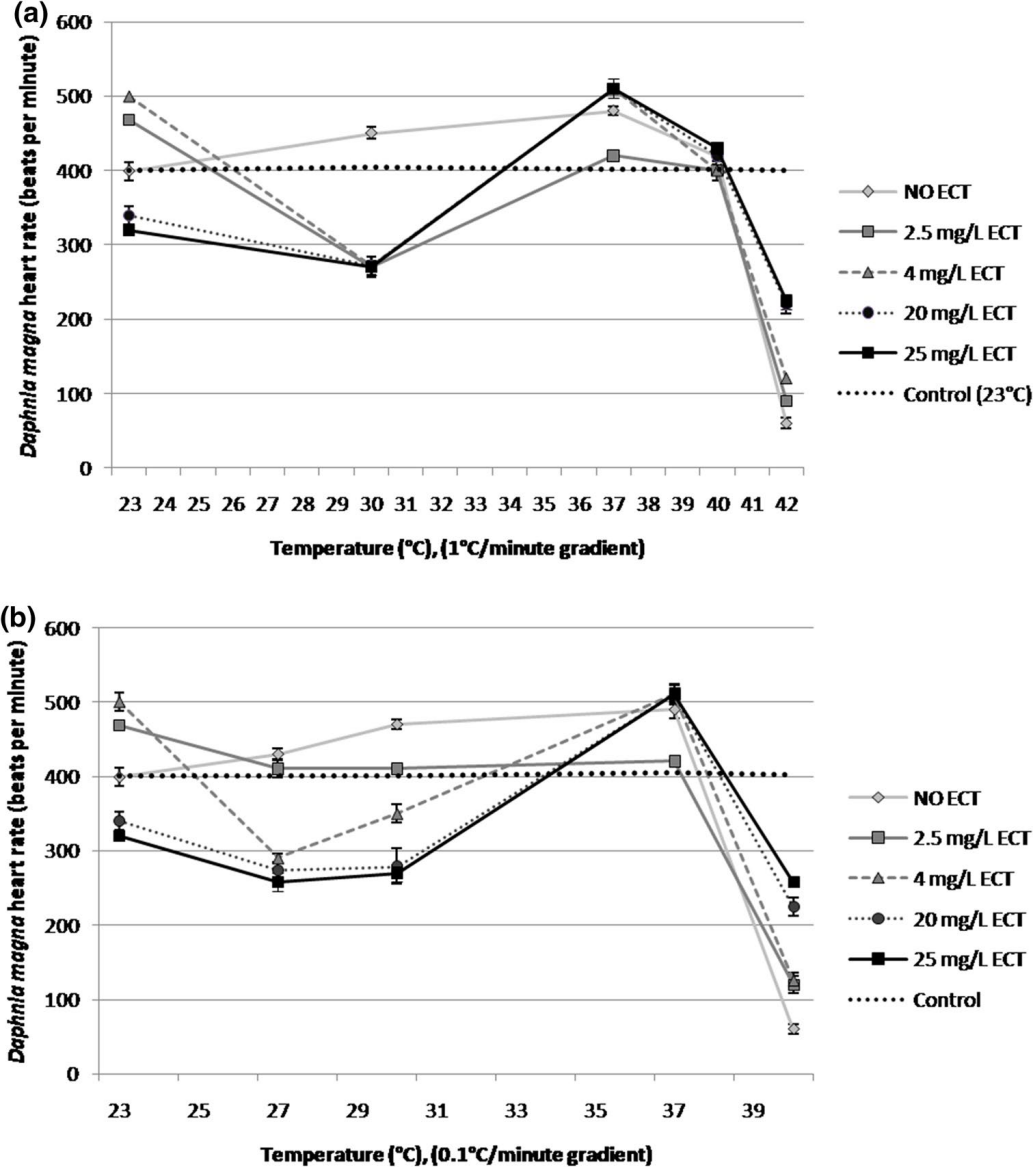
Heart rate of *Daphnia magna* exposed to various concentrations of ECT and subjected to heat stress with two gradients: 1 °C/min (**a**) and 0.1 °C/min (**b**). The *dashed*, *horizontal line* represents heart rate for control daphnids (at 23 °C) which were not heat stressed and not ECT-exposed. The results are presented as mean ± SD, *n* = 30

ECT showed modulatory influence on heart rate of daphnids at a temperature of 23 °C. Stimulation of heart rate (468 ± 6, 500 ± 12 bpm) was seen at concentrations of 2.5 and 4 mg/L, respectively when compared to the unexposed group of animals (399 ± 12 bpm). On the other hand, reduction of heart rate was observed at higher concentrations of ECT 20 and 25 mg/L with the values of 340 ± 12 and 320 ± 6 bpm, respectively. Heat stress with the temperature gradient of 1 °C resulted in transient decrease of heart rate at 30 °C in each experimental group of ECT-exposed animals but in a concentration-dependent manner with values of 270 ± 14, 272 ± 8, 272 ± 12 and 270 ± 10 bpm at 2.5, 4, 20 and 25 mg/L, respectively when compared to 400 ± 8 bpm in the control group of daphnids that were unexposed and not subjected to heat stress (Fig. 4a). Raising temperature to 37 °C increased the heart rate of ECT-exposed and the unexposed daphnids. However, ECT-exposed individuals increased their heart rate to 420 ± 6, 510 ± 12, 510 ± 6 and 510 ± 13 bpm at 2.5, 4, 20 and 25 mg/L of ECT, respectively. Daphnids that were heat-stressed but not treated with ECT showed stimulated heart rate (480 ± 6 bpm and 420 ± 5 bpm at 37 and 40 °C, respectively). Heating to 40 °C resulted in the inhibition of heart rate in ECT-treated individuals to 400 ± 13 (at 2.5 and 4 mg/L), 420 ± 12 and 430 ± 7 bpm at 20 and 25 mg/L, respectively. Continuation of heat stress with increasing temperature to 42 °C caused a rapid decrease of heart rate in the unexposed daphnids (60 ± 7 bpm). On the other hand, individuals exposed to ECT showed higher heart rates of 90 ± 6, 120 ± 14, 220 ± 12 and 224 ± 10 bpm at 2.5, 4, 20 and 25 mg/L of ECT, respectively. Heat stress with the temperature gradient of 0.1 °C also resulted in initial decrease of heart rate in ECT-treated daphnids at temperature of 27 °C (Fig. 4b). Daphnids exposed to ECT at concentrations of 4, 20 and 25 mg/L showed heart rates of 290 ± 12, 274 ± 6 and 258 ± 10 bpm in comparison to the unexposed or control daphnids (400 ± 7 bpm). However, daphnids treated with 2.5 mg/L ECT and the unexposed daphnids showed slightly stimulated heart rate (410 ± 6 and 430 ± 12 bpm, respectively). Further heat stress at 37 °C increased heart rate in ECT-exposed daphnids treated with ECT at 4, 20 and 25 mg/L to 510 ± 14, 511 ± 12 and 510 ± 10 bpm, respectively. Rapid decrease of heart rate of both ECT-exposed and unexposed daphnids was seen at 40 °C, however ECT attenuated the inhibition of heart rate to 120 ± 11, 126 ± 10, 225 ± 12 and 258 ± 6 bpm at concentrations of 2.5, 4, 20 and 25 mg/L, respectively, in comparison to 60 ± 8 bpm in the group of the unexposed animals.

### Thoracic limb movement

**Fig. 5:**
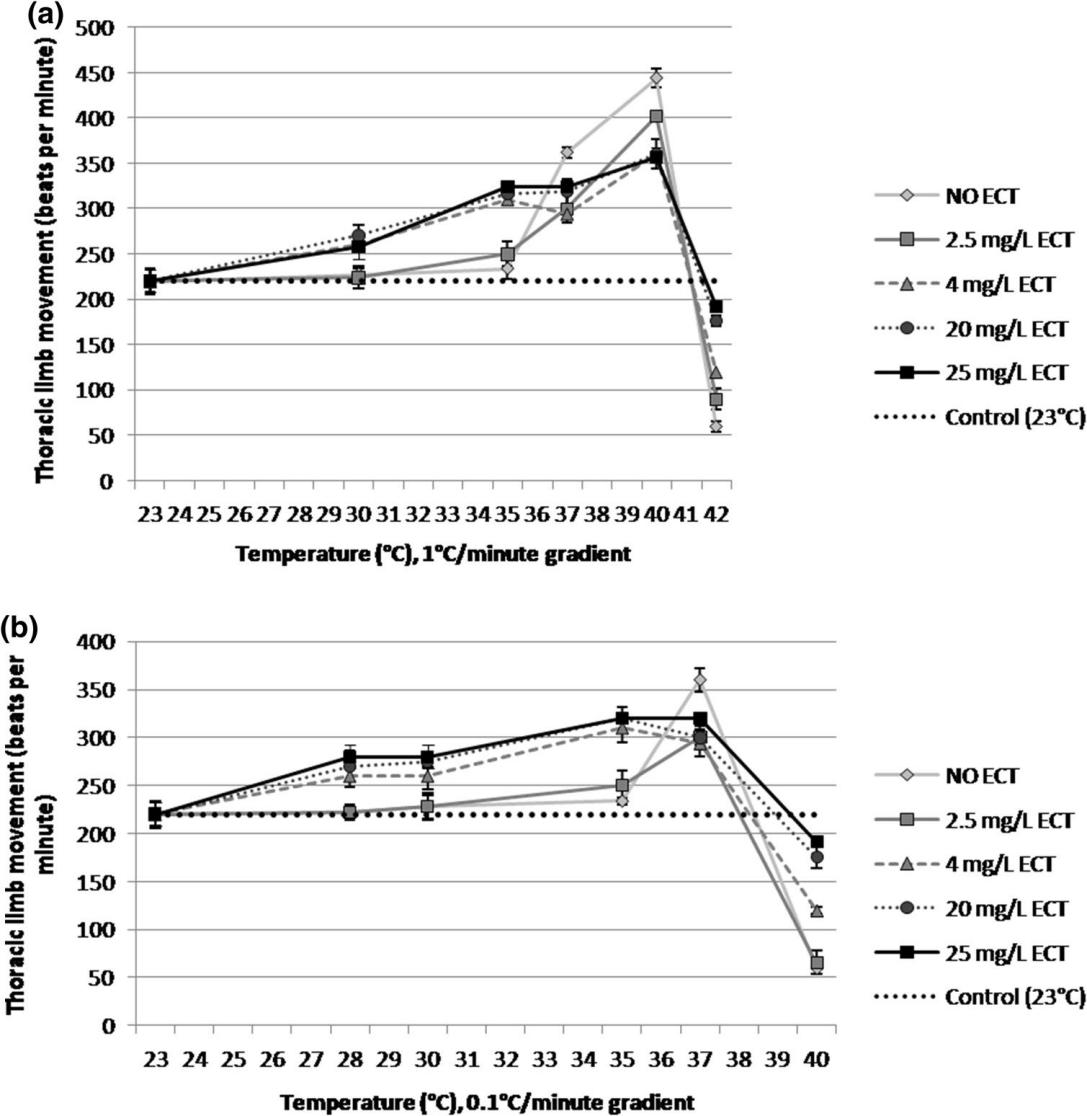
Thoracic limb activity in *Daphnia magna* treated with various concentrations of ecotine and subjected to heat stress in two temperature gradients: 1 °C and 0.1 °C/min. The *dashed*, *horizontal line* represents thoracic limb activity for control daphnids (at 23 °C) which were not heat stressed and not ECT-exposed. The results are presented as mean ± SD, *n* = 30

Thoracic limb activity in ECT-exposed and unexposed daphnids subjected to heat stress in both temperature gradients was altered. The unexposed, heat-stressed daphnids with the temperature gradient of 1 °C/min showed stimulation of thoracic limb movement at 37 °C (362 ± 6 bpm) in comparison to the control daphnids at 23 °C (228 ± 9 bpm) (Fig. 5a). The activity was stimulated with increasing temperature reaching maximum at 40 °C (444 ± 10 bpm) and a rapid decrease to 60 ± 6 bpm occured at 42 °C. Daphnids exposed to 25 mg/L that were subjected to heating showed increased activity of thoracic limbs with values of 258 ± 14 bpm at 30 °C, 324 ± 6 bpm at 35 and 37 °C, 354 ± 12 bpm at 40 °C, respectively and a decrease of limb activity at 42 °C to 192 ± 5 bpm. Similar effects were observed in heat-stressed daphnids exposed to lower concentrations of ECT, however the effects were less pronounced at lower temperature 30–37 °C and the increase of limb activity was less attenuated.

Heat-stressed daphnids at 30 °C with the temperature gradient of 0.1 °C/min exposed to 4, 20 and 25 mg/L of ECT showed higher limb activity (260 ± 15, 275 ± 12 and 280 ± 6 bpm, respectively) than that in the group of the unexposed daphnids (228 ± 4 bpm) (Fig. 5b). Significantly increased activity of limbs at 37 °C (360 ± 12 bpm) was found in the unexposed daphnids. However, the increase of limb activity in daphnids treated with ECT was less significant at four concentrations used than that in the non-treated daphnids and was 300 ± 7, 294 ± 14, 300 ± 12 and 320 ± 6 bpm at 2.5, 4, 20 and 25 mg/L of ECT, respectively. At 40 °C, limb movement frequency of the unexposed daphnids was 60 ± 6 bpm. On the other hand, daphnids treated with ECT at 42 °C showed increased limb activity of 120 ± 5, 176 ± 12 and 192 ± 4 bpm at 4, 20 and 25 mg/L of ECT, respectively.

### NOx level

**Fig. 6:**
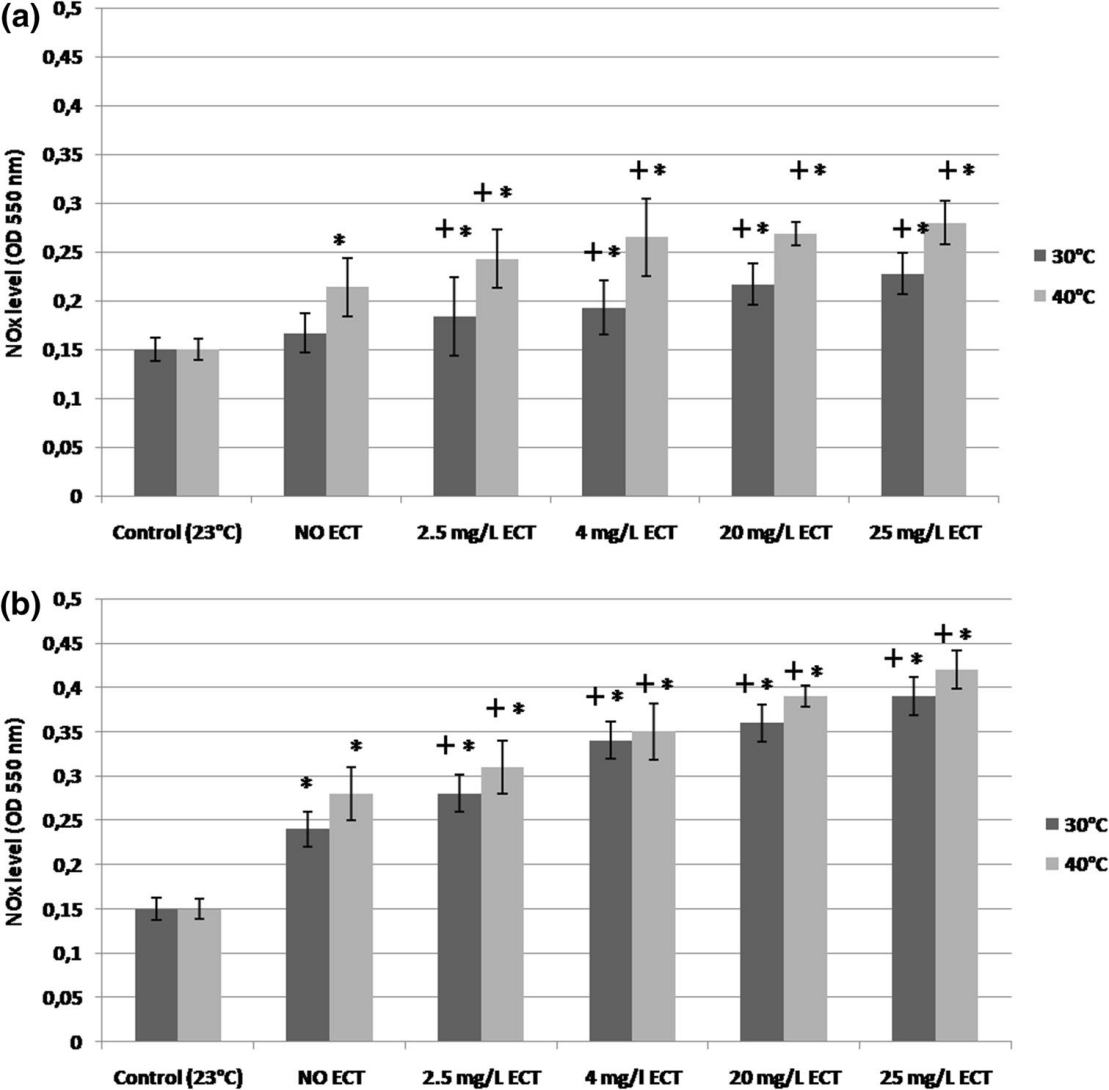
NOx production in *Daphnia magna* subjected to heat stress in the temperature gradient of 1 °C/min (**a**) and 0.1 °C/min (**b**) and at 30 and 40 °C and exposed to various concentrations of ECT. Control daphnids were not heat stressed and were not ECT-exposed. The results are presented as means ± OD, *n* = 30, *––statistical significance *p* < 0.05. Symbol “+” means statistical significance between ECT-treated and untreated daphnids.OD––optical density

The non-treated but heat-stressed daphnids in the temperature gradient of 1 °C/min showed increased production of NOx (OD = 0.21 ± 0.05) at 40 °C in a concentration-dependent manner when compared to not heated and not treated control individuals (OD = 0.15 ± 0.02), (Fig. 6a); however, the difference of NOx level between animals exposed to different concentrations of ECT was not very significant, especially at 30 °C. The highest stimulation of NOx production was noted in heat-stressed daphnids at 40 °C exposed to a concentration of 25 mg/l of ECT with OD value of 0.265 ± 0.02. Less pronounced but statistically significant alterations of NOx production were noted in daphnids taken for the assay at 30 °C. Heat stress in the temperature gradient of 0.1 °C/min resulted in the production of higher levels of NOx by daphnids in comparison to the individuals subjected to that of 1 °C/min (Fig. 6b). The highest levels of NOx were found at 40 °C in heat-stressed daphnids exposed to 25 mg/L of ECT (OD = 0.42 ± 0.02) in comparison to heat-stressed but unexposed animals (OD = 0.28 ± 0.03). Lower but statistically significant increase of NOx level was seen in the animals exposed to 20 and 4 and 2.5 mg/L of ECT with OD of 0.39 ± 0.012 and 0.35 ± 0.012 and 0.31 ± 0.03.

### Catalase activity

**Fig. 7:**
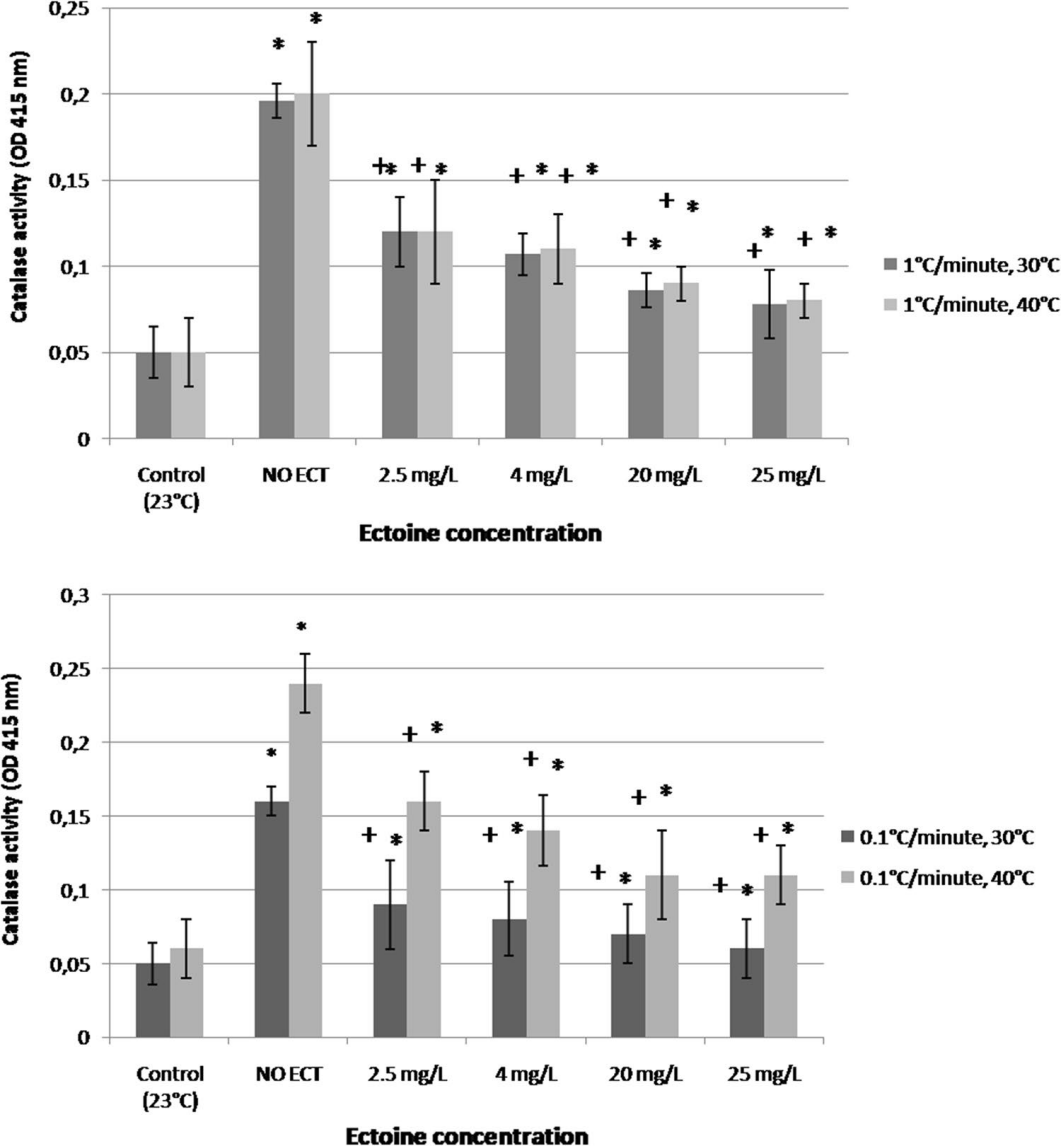
Catalase activity in *Daphnia magna* exposed to various concentration of ectoine at 30 °C and 40 °C of heat stress in the temperature gradient of 0.1 °C/min and at 40 °C at the temperature gradient of 1 °C. Control daphnids were not heat stressed and were not ECT-exposed. Results are presented as mean ± SD, *n* = 30, *asterisk* statistical significance *p* < 0.05. Symbol *plus* means statistical significance between ECT-treated and untreated daphnids. OD-optical density

Catalase activity was determined at 30 and 40 °C of heat stress with the temperature gradient of 0.1 °C/min and at 40 °C with that of 1 °C/min. A significant reduction of catalase activity was observed in heat-stressed daphnids exposed to ECT at 30 and 40 °C in a concentration-dependent manner (Fig. 7). Heat stress with the temperature gradient of 1 °C/min resulted in reduction of catalase activity in the ECT-exposed daphnids at 40 °C when compared to the ECT-free group. ODs of the enzyme activity at that temperature were 0.2 ± 0.01, 0.12 ± 0.02, 0.11 ± 0.02 0.09 ± 0.01, 0.08 ± 0.02 in ECT-free group and at ECT concentrations of 2.5, 4, 20 and 25 mg/L, respectively. The least pronounced increase of catalase activity was seen in daphnids exposed to 25 mg/L of ECT at 40 °C of heat stress in the temperature gradient of 0.1 °C/min (OD = 0.06 ± 0.02) in comparison to the unexposed individuals (OD = 0,16 ± 0,01). Catalase activity was also reduced at concentrations of 20 and 2.5 mg/L of ECT with OD of 0.07 ± 0,01 and 0.09 ± 0.02. ECT-exposed daphnids taken for the assays at 30 °C also showed a reduced activity of catalase in comparison with those of the unexposed animals, however the OD values were lower than those at 40 °C.

### HSP70 1A level

**Fig. 8:**
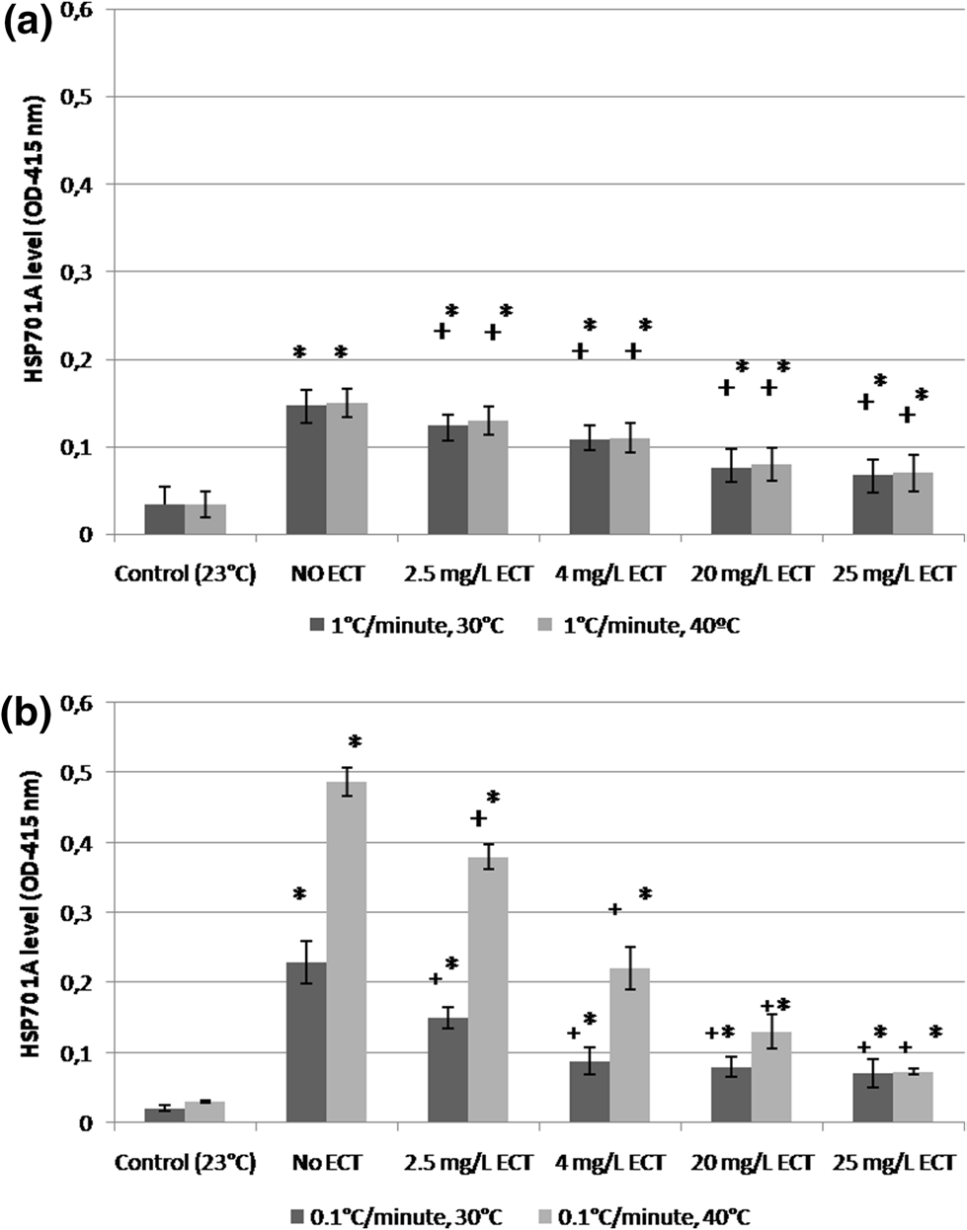
Level of heat shock protein, HSP70A1A in *Daphnia magna* exposed to ECT and subjected to heat stress. Determination of HSP70(A1A) activity was done in two temperature gradients: 0.1 (**a**) and 1 °C/min (**b**). Hsp70(A1A) level was determined at 30 and 40 °C of heat stress, in daphnids exposed to the temperature gradient of 0.1 °C/min gradient and at 40 °C of the stress in 1 °C/min. Control daphnids were not heat stressed and not exposed to any concentration of ECT. Results are presented as mean ± SD, *n* = 30. *Asterisk* statistical significance to the control *p* < 0.05, Symbol *plus* means statistical significance between ECT-treated and untreated daphnids. OD-optical density

Heat stress in the temperature gradient of 0.1 °C/min increased the level of HSP70 1A at 30 and 40 °C in both ECT-treated and not treated daphnids in comparison to the control daphnids maintained at room temperature (Fig. 8a). However, the animals exposed to ECT showed a significant and concentration-dependent decrease of the HSP70 1A concentration, when compared to not treated but heat-stressed daphnids. The highest decrease of HSP70 1A production during heat stress at 40 °C was observed at 25 mg/L of ECT (OD = 0.073 ± 0.004) in comparison to the group of not exposed animals (OD = 0.49 ± 0.02). The least pronounced increase of the protein production was also seen at 4 mg/L of ECT (OD = 0.22 ± 0.03). Alterations of HSP70 1A concentration in the presence of ECT was also detected in daphnids examined at a temperature of 30 °C of heat stress but changes were less significant than those in daphnids tested at 40 °C. Heating experiment with the temperature gradient of 1 °C/min showed slight differences in HSP70 1A concentration at 40 °C in ECT-exposed and unexposed daphnids (Fig. 8b). The heat stressed but not treated daphnids showed increase of HSP70 1A concentration (0.15 ± 0.03), however it was much lower that that in the animals heat stressed in the gradient of 0.1 °C/min. The decreased HSP70 1A level was seen at all concentrations of ECT: 2.5 (0.13 ± 0.012), 4 mg/L (OD = 0.11 ± 0.016), 20 (OD = 0.08 ± 0.019) and 25 mg/L (OD = 0.07 ± 0.017), and in in comparison to ECT-free group (OD = 0.15 ± 0.018) and was not altered in comparison to that measured at 30 °C.

## Discussion

To our best knowledge, this is the first study showing protective influence of ECT on survival, behavioural, physiological and biochemical parameters of *Daphnia magna* during heat stress. Effects of hyperthermia on survival of *Daphnia magna* were previously described by some authors (Kivuori and Lahdes 1996; Lagerspetz, 2000); however, no results on protective effects of ECT on heat-affected animals are available. The results of the present study showed that survival during recovery of untreated daphnids subjected to heat stress was decreased when compared to that of ECT-treated daphnids. Reduction of survival of daphnids subjected to heat stress was also observed by Kivuori and Lahdes (1996). Similarly to our results, the authors observed a higher reduction of survival time of daphnids previously subjected to heat stress with higher upper limit of temperature. However, survival times of daphnids used in the present study were much shorter since we used gradients with higher value of upper limit of temperature which probably caused more detrimental effects to the enzymatic machinery in the immobilised daphnids. We indicated that time to immobilisation of heated daphnids was prolonged in the presence of ECT even at its low concentrations. The protective effect of this amino acid seems to be concentration-dependent, and more distinct in the temperature gradient of 0.1 °C than 1 °C/min. Our study showed that ECT prolonged survival of daphnids during recovery after heat stress. After termination of heating the immobilised daphnids previously treated with ECT regained temporal ability to swim. However, daphnids without thermal protection of ECT showed highest mortality among all heat-stressed. Higher mortality rate was found in the group of daphnids subjected to heating with the slower temperature gradient. Higher survival rate was seen in ECT-exposed dapnids, however significant thermal defects did not allow them to survive more than 24 h.

### Swimming behaviour

Our studies revealed that increase of swimming speed of *Daphnia magna* induced by higher temperature. This change of behaviour may be a result of heat avoidance which was also observed by some authors (Lagerspetz, 2000; Paul et al., 2004). We showed that daphnids that were not exposed to ECT but subjected to heat stress showed a significant increase of their swimming velocity at a temperature of 37 °C. More distinct escaping response was observed in daphnids subjected to the temperature gradient of 1 °C/min compared to those heated with that of 0.1 °C/min. Protective effects of ECT on daphnids subjected to heat stress was manifested by a reduced escaping response of ECT-exposed daphnids at 37 and 40 °C compared to that of unexposed animals. Heat stress induced with the slower gradient resulted in a rapid reduction of swimming velocity of both ECT-treated and non-treated daphnids at 40 °C; however, ECT slightly attenuated the velocity drop. Differences of swimming speed between ECT-treated and not exposed daphnids were more distinct when the animals were stressed with the faster temperature gradient. This is opposite to our findings associated with the other endpoints determined in this study showing more significant changes during heat stress with the slower temperature gradient. It suggests that swimming velocity of aquatic animals is a very reliable and quick behavioural biomarker of heat stress. On the basis of our results from behavioural studies we conclude that better thermoprotection may be obtained with the use of higher concentrations of ECT since only daphnids that were unexposed or treated to the lowest concentrations of ECT showed increased swimming velocity during hyperthermia. The molecular mechanism of ECT protective effects of swimming velocity is not known and further studies are required to elucidate it.

### Heart rate and thoracic limb activity

*Daphnia magna* is a senstitive crustacean frequently used as a model organism for optical assessment of the effect of various compounds on physiological parameters (Villegas-Navarro et al., 2003; Campbell et al., 2004). Since this animal is transparent, physiological parameters, such as heart activity and thoracic limb movements can be measured with non-invasive methods such as light microscopy. *Daphnia* *magna* heart activity is temperature-dependent and its rate increases at higher and decreases at lower temperature. It is also altered by different chemicals (Campbell et al., 2004; Paul et al., 2004). It is known that temperature changes may alter metabolic rate, cardiac and enzymatic activity and protein metabolism in daphnids (Schwerin et al., 2009). Biochemical reactions in those organisms are faster at higher temperatures. As metabolic rate increases, cells have higher oxygen demands. In a consequence, heart beat and thoracic limbs stimulate their activity in order to provide more oxygen (Pirow et al., 1999b). However, at a temperature of 40 °C when enzymes are denatured metabolic rate rapidly decreases. Our study showed that the unexposed heat-stressed daphnids showed increase of heart rate caused by increase of metabolism at higher temperature. However, subsequent rapid decrease of heart rate caused by enzyme denaturation. ECT-treated daphnids demonstrated modulatory activity of the amino acid on heart rate heat-stressed daphnids. Its inhibitory influence was observed in both gradients at lower temperature (27–30 °C), however at higher temperature (above 30 °C) ECT-induced stimulation of heart rate particularly at 37 °C. Interestingly, the increased heart rate was also seen in daphnids with maintained ability to swim at critical temperature prior to immobilisation. Such alteration of heart rate during the heat stress may be a result of temperature-dependent modulation of metabolic activity induced by ECT. Stimulation of heart rate may result.

Thoracic limb movement is a common physiological marker of *Daphnia magna* metabolic activity and its rate may be altered by various environmental stressors (Pirow et al., 1999a; Penalva-Arana et al., 2011). Heat stress of *Daphnia magna* results in initial stimulation of thoracic limb activity and its subsequent decrease at sublethal temperature (Paul et al., 2004). Heat stress increases metabolic rate and oxygen demands in poikilothermic organisms. Therefore, activity of thoracic limbs which play a very important role in ventilatory processes and gas exchange is increased. Our study showed the heat stress-induced alterations of limb activity in daphnids subjected to hyperthermia but which were not treated with ECT. The activity of limbs was increased at the lower range of temperature (30–35 °C), however it was maintained approximately on the same level even when temperature increased to 40 °C which suggests that ECT slightly stimulates the activity of limbs at higher temperature and attenuates its rapid changes at its critical values prior to animal immobilisation. Increased activity of the limbs at the highest concentrations may be explained by ECT-induced protection of enzymes from heat-induced denaturation. The results clearly demonstrate the stabilising effects of ECT on heart during heat stress.

### NOx level

NO is a signalling molecule playing many physiological roles in organisms. (Regulski and Tully 1995; Demenge and Ribuot 2001). It is known that nitric oxide at low concentrations may have antioxidant capacity, however its high concentrations may induce oxidative damage to cells (Borniquel et al., 2006). Some results indicate that heat stress stimulates NO production in animals but also in bacteria and plants (Arnaud et al., 2011; Yarullina et al., 2010). It seems that production of NOx in daphnids subjected to higher temperature in the presence of ECT is probably associated with their response to oxidative stress. This molecule is essential in thermoregulation processes Steiner and Branco 2001; Sanches et al., 2002). Its osmoregulatory and excretory functions were characterised in marine invertebrates and insects (; Davies, 2000; Palumbo, 2005). This molecule is a very unstable compound in the in vitro conditions, therefore its derivatives nitrites and nitrates (surrogate biomarkers) are usually measured to evaluate its level. Studies performed on mammals showed that hyperthermia induces vasodilation with induction of NO production. Some authors found that production of heat-related molecules, like heat shock proteins, (HSP96 and HSP70) activate the release of NO from antibody presenting cells (Panjwani et al. 2002) which may play some thermoprotective roles. It has been reported that overproduction of this molecule may lead to mutagenesis and death (Wink et al. 1991), however it was also shown that at low concentrations it induces cytoprotective effects against reactive oxygen species (Wink et al., 1993). Our studies showed a slight induction of NOx in the unexposed daphnids during heat stress, however, its higher levels were found in daphnids exposed to ECT in the slower temperature gradient which were tested at 40 °C. Slow rise of temperature-induced less disturbances in biochemical processes which allowed producing more NOx. Additionally, since higher level of NOx was noted in more heat-resistant daphnids after they were treated with higher concentrations of ECT, it may suggest a possible role of NOx in the mechanism of thermoprotective effects of ECT in daphnids.

### Catalase activity

Heat stress increases the level of oxygen radicals probably by the destruction of the electron transport assemblies of the cell membrane and induces production of antioxidative enzymes (Ando et al., 1997; Jia et al., 2011). CAT is a very essential antioxidant enzyme protecting aerobic organisms against oxidative damage and has been used as a biomarker of oxidative stress in *Daphnia magna* (Kim et al., 2010). The activity of CAT was shown to be increased under heat stress in response against high temperature-induced damage of proteins or cell membranes (Currie et al., 1988; Steare and Yellon 1994). Some authors determined the level of CAT in *Daphnia magna* during hyperthermia combined with other stressors (Muyssen et al., 2010; Zeis et al. 2013). Our studies showed that the level of CAT produced by heat-subjected but ECT-unexposed daphnids was increased in comparison to that of daphnids remaining at room temperature. The level of the enzyme raised with increasing temperature in daphnids subjected to heating with the gradient of 0.1 °C/min. ECT seems to have antioxidative properties during hyperthermia in daphnids since heat stress resulted in production of lower level of CAT in ECT-exposed daphnids in comparison to the unexposed ones. The protective effect was concentration-dependent and was inhibited with increasing temperature and longer duration of heat stress. ECT-induced inhibition of CAT production suggests its protective action against oxidative response in crustaceans subjected to high temperature.

### HSP70 1A level

Heat shock proteins found in various organisms with several functions are a part of cell repair system and they are produced in response to hyperthermia or other environmental stressors (Kregel, 2002). This family includes proteins of different molecular weight and some of them may serve as chaperone maintaining proper conformation of cellular proteins and preventing against unfolding or misfolding and aggregation. Heat shock proteins were described in *Daphnia magna* subjected to different stressful conditions (Chen et al.,^1999^; Pauwels et al. 2007; Haap et al., 2008). Induction of HSP70 1A in *Daphnia magna* after the exposure to diclofenac and dimethyl sulfoxide was described by Haap et al. (2008). Production of HSP 60, 70 and 90 kDa in those crustaceans was also observed during heat stress (Mikulski et al., 2009; 2011). Our study showed that the induction of HSP70 1A was evident in daphnids subjected to heat stress but which were not treated with ECT. More significant production of HSP70 1A was seen in the temperature gradient of 0.1 °C/min. It suggests that longer period of heat stress even with slower temperature gradient induces higher level of HSP70 1A than shorter heat stress but with the faster gradient. Higher production of HSP70 1A in the slower gradient may result from longer period of time (about 4 times) of protein biosynthesis compared to the faster gradient until reaching point of protein denaturation at 40 °C. Moreover, heat stress in slower gradient characterised by less rapid changes of temperature for longer period of time induces less severe disturbances in the process of protein production (for example on the level of transcription or translation), therefore higher amount of HSP70 1A can be produced. We showed that ECT inhibits the production of HSP70 1A in *Daphnia magna* during heat stress. The level of HSP70 1A was significantly less induced in heat-stressed daphnids that were exposed to ECT and were able to survive much longer in comparison to the ECT-nonexposed individuals. It suggests that protective effects of ectoine on survival of *Daphnia magna* subjected to heat stress correlates with the reduction of heat shock protein HSP70 1A level. Since in ECT-exposed daphnids production of HSP70 1A was inhibited, we can suppose that ECT might act similarly to heat shock protein HSP70 1A stabilising cell membranes and enzymes at higher temperature, and, despite high temperature, induction of those proteins was inhibited. On the other hand, some authors found expression of hsp70 and hsp70B′ genes in heat-stressed isolated human keratinocytes exposed to ECT (Buommino et al., 2005). The discrepancy between our findings and results obtained by the authors may be explained by different experimental conditions (invertebrate animals versus mammalian cells or in vivo versus in vitro) and by the fact that ECT used at high concentrations may itself be stressful to keratinocytes and in a consequence induce production of HSPs. Protective effects of ECT in daphnids do not seem to be associated with the induction of HSP70 1A. HSP70 1A may be less induced by temperature in the presence of ECT and/or ECT may itself act as heat shock agent in microcrustaceans.

Our study showed protective effects of ectoine on the survival and behavioural, physiological and biochemical levels in *Daphnia magna*. Mechanisms of thermoprotective action of ECT in daphnids do not seem to be associated with HSP70 kDa induction since its levels were less stimulated in ECT-treated daphnids subjected to heating. It is known that ECT stabilises the cell membrane protein and inhibits their denaturation at high temperatures by maintaining their structures towards folded conformations and preventing their unfolding. Thermoprotection of ECT may be a additive effect of several changes such as increased cell membrane protein stabilisation and its enhanced fluidity and attenuation of enzyme denaturation, however full elucidation of its mechanisms of its action requires further studies.

## References

[CR1] American Society of Testing and Materials (1986). Standard practice for conducting static acute toxicity tests on wastewaters with Daphnia: annual book of ASTM standards. Philadelphia.

[CR2] Ando M, Katagiri K, Yamamoto S, Wakamatsu K, Kawahara I, Asanuma S, Usuda M, Sasaki K (1997). Age-related effects of heat stress on protective enzymes for peroxides and microsomal monooxygenase in rat liver. Environ Health Perspect.

[CR3] Arnaud C, Laubriet A, Joyeux M, Godin-Ribuot D, Rochette L, Penalva-Arana DC, Forshay-Johnson KPTJ, Strickler JR, Dodson SI (2011). Chytrid infection reduces thoracic beat and heart rate of *Daphnia pulicaria*. Hydrobiologia.

[CR4] Borniquel S, Valle I, Cadenas S, Lamas S, Monsalve M (2006). Nitric oxide regulates mitochondrial oxidative stress protection via the transcriptional coactivator PGC-1. FASEB J.

[CR5] Buommino E, Schiraldi C, Baroni A, Paoletti I, Lamberti M, De Rosa M, Tufano MA (2005). Ectoine from halophilic microorganisms induces the expression of hsp70 and hsp70B’ in human keratinocytes modulating the proinflammatory response. Cell Stress Chap.

[CR6] Campbell AK, Wann KT, Matthews SB (2004). Lactose causes heart arrhythmia in the water flea *Daphnia pulex*. Comp Biochem Physiol Part B.

[CR7] Chen CY, Sillett KB, Folt CL, Whittemore SL, Barchowsky A (1999). Molecular and demographic measures of arsenic stress in *Daphnia pulex*. Hydrobiologia.

[CR8] Currie RW, Karmazyn M, Kloc M, Mailer K (1988). Heat-shock response is associated with enhanced postischemic ventricular recovery. Circ Res.

[CR9] Davies S (2000). Nitric oxide signalling in insects. Insect Biochem Mol Biol.

[CR10] Demenge P, Ribuot C (2001). Role of nitric oxide synthases in the infarct size-reducing effect conferred by heat stress in isolated rat hearts. Brit J Pharmacol.

[CR11] Galinski EA, Pfeiffer HP, Truper HG (1985). 1, 4, 5, 6-Tetrahydro-2-methyl-4-pyrimidinecarboxylic acid. A novel cyclic amino acid from halophilic phototrophic bacteria of the genus Ectothiorhodospira. Eur J Biochem.

[CR12] Göller K, Galinski EA (1999). Protection of a model enzyme (lactate dehydrogenase) against heat, urea and freeze-thaw treatment by compatible solute additives. J Mol Cat B.

[CR13] Goth L (1991). A simple method for determination of serum catalase activity and revision of reference range. Clin Chim Acta.

[CR14] Griess P (1879). Bemerkungen zu der abhandlung der H.H. Weselsky und Benedikt “Ueber einige azoverbindungen”. Chem Ber.

[CR15] Haap T, Triebskorn R, Köhler HR (2008). Acute effects of diclofenac and DMSO to *Daphnia magna*: immobilisation and hsp70-induction. Chemosphere.

[CR16] Harishchandra RK, Wulff S, Lentzen G, Neuhaus T, Galla HJ (2010). The effect of compatible solute ectoines on the structural organization of lipid monolayer and bilayer membranes. Biophys Chem.

[CR17] Heugens EH, Jager T, Creyghton R, Kraak MH, Hendriks AJ, van Straalen NM, Admiraal W (2003). Temperature-dependent effects of cadmium on *Daphnia magna*: accumulation versus sensitivity. Environ Sci Technol.

[CR18] Jia FX, Dou W, Hu F, Wang JJ (2011). Effects of thermal stress on lipid peroxidation and antioxidant enzyme activities of oriental fruit fly, *Bactrocera dorsalis* (Diptera; Tephritidae). Florida Entomol.

[CR19] Kim J, Kim S, An KW, Choi CY, Lee S, Choi K (2010). Molecular cloning of *Daphnia magna* catalase and its biomarker potential against oxidative stresses. Comp Biochem Physiol C: Toxicol Pharmacol.

[CR20] Kivuori LA, Lahdes EO (1996). How to measure the thermal death of *Daphnia*? A comparison of different heat tests and effects of heat injury. J Therm Biol.

[CR21] Knapp S, Ladenstein R, Galinski EA (1999). Extrinsic protein stabilization by the naturally occuring osmolytes b-hydroxyectoine and betaine. Extremophiles.

[CR22] Kregel KC (2002). Invited review: heat shock proteins: modifying factors in physiological stress responses and acquired thermotolerance. J Appl Physiol.

[CR23] Lagerspetz KYH (2000). Thermal avoidance and preference in *Daphnia magna*. J Therm Biol.

[CR24] Lippert K, Galinski EA (1992). Enzyme stabilization by ectoine-type compatible solutes: protection against heating, freezing and drying. Appl Microbiol Biotechnol.

[CR25] Mikulski A, Grzesiuk M, Kloc M, Pijanowska J (2009). Heat shock proteins in *Daphnia* detected using commercial antibodies: description and responsiveness to thermal stress. Chemoecology.

[CR26] Mikulski A, Bernatowicz P, Grzesiuk M, Kloc M, Pijanowska J (2011). Differential levels of stress proteins (HSPs) in male and female *Daphnia magna* in response to thermal stress: a consequence of sex-related behavioral differences?. J Chem Ecol.

[CR27] Muyssen BTA, Messiaen M, Janssen CR (2010). Combined cadmium and temperature acclimation in *Daphnia magna*: physiological and sub-cellular effects. Ecotoxicol Environ Saf.

[CR28] Nagata S, Wang YB (2001). Accumulation of ectoine in the halotolerant brevibacterium sp. JCM6894. J Biosci Bioeng.

[CR29] Palumbo A (2005). Nitric oxide in marine invertebrates: a comparative perspective. Comp Biochem Physiol A.

[CR30] Panjwani NN, Popova L, Srivastava PK (2002). Heat shock proteins gp96 and hsp70 activate the release of nitric oxide by APCs. J Immunol.

[CR31] Pastor JM, Bernal V, Salvador M, Argandoña M, Vargas C, Csonka L, Sevilla Á, Iborra JL, Nieto JJ, Cánovas M (2013). Role of central metabolism in the osmoadaptation of the halophilic bacterium *Chromohalobacter salexigens*. J Biol Chem.

[CR32] Paul RJ, Lamkemeyer T, Maurer J, Pinkhaus O, Pirow R, Seidl M, Zeis B (2004). Thermal acclimation in the microcrustacean *Daphnia:* a survey of behavioural, physiological and biochemical mechanisms. J Therm Biol.

[CR33] Pauwels K, Stoks R, Decaestecker E, De Meester L (2007). Evolution of heat shock protein expression in a natural population of *Daphnia magna*. Am Nat.

[CR34] Penalva-Arana DC, Forshay K, Johnson PTJ, Strickler JR, Dodson SI (2011). Chytrid infection reduces thoracic beat and heart rate of *Daphnia pulicaria*. Hydrobiologia.

[CR35] Pinkhaus O, Schwerin S, Pirow R, Zeis B, Buchen I, Gigengack U, Koch M, Horn W, Paul RJ (2007). Temporal environmental change, clonal physiology and the genetic structure of a *Daphnia* assemblage (*D. galeata*-*hyalina* hybrid species complex). Freshw Biol.

[CR36] Pirow R, Wollinger F, Paul RJ (1999). The importance of the feeding current for oxygen uptake in the water flea Daphnia *magna*. J Exp Biol.

[CR37] Pirow R, Wollinger F, Paul RJ (1999). The sites of respiratory gas exchange in the planktonic crustacean Daphnia magna: an in vivo study employing blood haemoglobin as an internal oxygen probe. J Exp Biol.

[CR38] Regulski M, Tully T (1995). Molecular and biochemical characterization of dNOS: a Drosophila Ca^2+^/calmodulin-dependent nitric oxide synthase. Proc Nat Ac Sci USA.

[CR39] Roessler M, Müller V (2001). Osmoadaptation in bacteria and archaea: common principles and differences. Environ Microbiol.

[CR40] Sanches DB, Steinerb AA, Luiz GS (2002). Involvement of neuronal nitric oxide synthase in restraint stress-induced fever in rats. Physiol Behav.

[CR41] Santos H, da Cota MS (2002). Compatible solutes of organisms that live in hot saline environments. Environ Microbiol.

[CR42] Schwerin S, Zeis B, Lamkemeyer T, Paul RJ, Koch M, Madlung J, Fladerer C, Pirow R (2009). Acclimatory responses of the *Daphnia pulex* proteome to environmental changes. II. Chronic exposure to different temperatures (10 and 20 °C) mainly affects protein metabolism. BMC––Physiology.

[CR43] Shimizu N, Ogino C, Kawanishi T, Hayashi Y (2002). Fractal analysis of *Daphnia* motion for acute toxicity bioassay. Inc Environ Toxicol.

[CR44] Singer MA, Lindquist S (1998). Thermotolerance in *Saccharomyces cerevisiae*: the yin and yang of trehalose. Trends Biotechnol.

[CR45] Smith KL, Galloway TS, Depledge MH (2000). Neuro-endocrine biomarkers of pollution-induced stress in marine invertebrates. Sci Total Environ.

[CR46] Steare SE, Yellon DM (1994). Increased endogenous catalase activity caused by heat stress does not protect the isolated rat heart against exogenous hydrogen peroxide. Cardiovasc Res.

[CR47] Steiner AA, Branco LGS (2001). Nitric oxide in the regulation of body temperature and fever. J Therm Biol.

[CR48] Villegas-Navarro A, Rosas-L E, Reyes JL (2003). The heart of *Daphnia magna*: effects of four cardioactive drugs. Comp Biochem Physiol Part C.

[CR49] Wei YH, Yuan FW, Chen WC, Chen SY (2011). Production and characterization of ectoine by Marinococcus sp. ECT1 isolated from a high-salinity environment. J Biosci Bioeng.

[CR50] Wink DA, Kasprzak KS, Maragos CM, Elespuru RK, Misra M, Dunams TM, Cebula TA, Koch WH, Andrews AW, Allen JS, Keefer LK (1991). DNA deaminating ability and genotoxicity of nitric oxide and its progenitors. Science.

[CR51] Wink DA, Hanbauer I, Krishna MC, DeGraff W, Gamson J, Mitchell JB (1993). Nitric oxide protects against cellular damage and cytotoxicity from reactive oxygen species. Proc Natl Acad Sci USA.

[CR52] Yarullina DR, Smolentseva OA, Kolpakov AI, Ilinskaya ON (2010). High temperature stress activates nitric Oxide synthesis in *Lactobacillus plantarum*. Dokl Biol Sci.

[CR53] Zeis B, Becker D, Gerke P, Koch M, Paul RJ (2013). Hypoxia-inducible haemoglobins of *Daphnia pulex* and their role in the response to acute and chronic temperature increase. BBA Protein Proteomic.

[CR54] Zhang L, Wang Y, Zhang C, Wang Y, Zhu D, Wang C, Nagata S (2006). Supplementation effect of ectoine on thermostability of phytase. J Biosci Bioeng.

